# Focus issue on plant immunity: from model systems to crop species

**DOI:** 10.3389/fpls.2015.00195

**Published:** 2015-03-26

**Authors:** Benjamin Schwessinger, Rebecca Bart, Ksenia V. Krasileva, Gitta Coaker

**Affiliations:** ^1^Department of Plant Pathology, University of CaliforniaDavis, CA, USA; ^2^Lawrence Berkeley National Laboratory, Joint BioEnergy Institute and Physical Biosciences DivisionBerkeley, CA, USA; ^3^Donald Danforth Plant Science CenterSt. Louis, MO, USA; ^4^The Genome Analysis Centre, Norwich Research ParkNorwich, UK; ^5^The Sainsbury Laboratory, Norwich Research ParkNorwich, UK

**Keywords:** crops, agricultural, innate immunity, plant pathology, translational research, immune signaling

One of the largest challenges of our time is to enhance agricultural production to feed a growing population in the midst of a changing climate. According to estimates, the global population will increase from 7 to 9 billion people by 2050 requiring a 60% increase in food in order to meet demand (Alexandratos and Bruinsma, 2012). Only the combination of reduction of food waste together with an increase in food productivity will enable us to meet this daunting challenge (Godfray et al., 2010). Advancements in agricultural practices, technology, food transport, and crop yields on marginal lands will be required to address this looming food production challenge. Crop losses due to plant disease significantly impact agriculture, with ~15% of global crop production lost due to preharvest plant disease (Pinstrup-Andersen, 2001; Oerke, 2006). Studies of model plants, such as *Arabidopsis*, have significantly enhanced our understanding of plant innate immune perception and signaling. For example, the identification of classical plant resistant genes in *Arabidopsis* and other model dicots facilitated the successful cloning of multiple wheat rust resistant genes (Ellis et al., 2014; Wulff and Moscou, 2014). With advancements in genome sequencing and analyses, we are now at a stage to exploit the basic knowledge gained in plant model species at a full genome scale in crops (Piquerez et al., 2014).

This Research Topic encompasses a collection of reviews, opinions, perspectives, as well as primary research articles investigating the interaction between crop plants and a variety of pathogens including fungi, bacteria, viruses and aphids (Ellis et al., 2014; Huet, 2014; Jaouannet et al., 2014; Nicaise, 2014; Stergiopoulos and Gordon, 2014; Wiesel et al., 2014). Immune receptors with extracellular domains can recognize pathogen proteins or conserved microbial signatures in the apoplast. Robinson and Bostock (2015) highlight the role of oomycete branched β-1,3-glucans and eicosapolyenoic acids for eliciting plant immune responses in a variety of plants. In light of recent research, these “elicitors” are likely conserved microbial signatures and can be reclassified as microbe associated molecular patterns (MAMPs) (Robinson and Bostock, 2015). Several articles reveal commonalities as well as differences in pathogen perception and signaling across diverse plant species (Barrios Perez and Brown, 2014; De Vleesschauwer et al., 2014; Figueiredo et al., 2014; Piquerez et al., 2014), highlighting the need for investigating innate immunity in wide array of plants with a focus on crop species. The same sets of hormones are critical for defense signaling in vascular plants. However, as illustrated by De Vleesschauwer et al. (2014) in their comparison of *Arabidopsis* and rice, the molecular mechanisms and the effect on the cross-talk between different hormones can vary significantly. Studies focusing on the molecular factors that define and limit pathogen host range as well as the ability to transfer non-host resistance (NHR) across plant species will significantly enhance scientist's ability to develop novel disease control strategies (Bettgenhaeuser et al., 2014; Stam et al., 2014). Rust fungi are a group of broadly distributed plant pathogens. However, distinct rust pathogens can exhibit very broad host range (*Phakopsora pachyrhizi*), narrow host range (*Puccinia triticina*) or a continuum between the two (Bettgenhaeuser et al., 2014). According to Stam et al. (2014), current evidence supports the hypothesis that in distantly related plant species NHR is mostly driven by the pathogens inability to suppress pattern recognition receptor mediated immunity at the plasma membrane. However, in closely related plant species NHR is mostly caused by the recognition of effector molecules by intracellular immune receptors containing nucleotide binding site and c-terminal leucine rich repeats (NLRs) (Stam et al., 2014). The interactions between different microorganisms, pathogenic and nonpathogenic, should also be considered when investigating plant defense and NHR, because interactions between plant colonizing organisms can impact disease development and outcome (Hale et al., 2014; Stergiopoulos and Gordon, 2014). When analyzing plant microbiomes, it will be important to complement survey studies with hypothesis-driven research, such as those outlined by Hale et al. (2014).

Parasitic plants, viruses as well as insects significantly impact agricultural production. Aphids cause significant crop losses by acting as a vector for plant viruses as well as causing damage due to feeding (Jaouannet et al., 2014). Jaouannet et al. (2014) highlight the importance of aphid-plant interactions as well as the role of classical plant immune receptors and signaling networks in recognizing these insect pathogens. Similarly, immunity against parasitic plants of the genus *Cuscuta* is hypothesized to employ the same classes of immune receptors yet knowledge of the molecular mechanisms involved during the infection process and the ensuing immune response are still sparse (Kaiser et al., 2015). In addition to these classic plant defense pathways, antiviral immunity involves several distinct RNA interference pathways that limit pathogen replication and spread (Nicaise, 2014).

We have entered an exciting era of research on plant-pathogen-microbe interactions. Large-scale genome sequencing of both plant and pathogen populations is now feasible and can help formulate multiple testable hypotheses. The “Integrated Decoy Hypothesis” posed by Dodds and colleagues was developed from a combination of wet lab experiments coupled with genome analyses to identify genetically linked NLR pairs, where one receptor has an additional “sensing” domain targeted by effectors (Cesari et al., 2014). This hypothesis can now be directly tested to determine if “sensor” NLRs are decoys for pathogen effectors or bona fide virulence targets using existing near isogenic lines (Wu et al., 2015). Conserved secretion signals have enabled the identification of thousands of candidate effectors in oomycete and bacterial pathogens. Pathogen genomes can also be mined to identify core effectors. These core effectors can be subsequently used to screen germplasms known to harbor resistance(s) to identify the recognized effector and cognate *NLR* gene. This knowledge of the molecular identities will enable the deployment of cultivars recognizing conserved pathogen effectors and monitoring their allelic variation in the field (Huet, 2014; Vasudevan et al., 2014; Wulff and Moscou, 2014). To date screens identifying *NLR* /effector pairs are still a major bottleneck in plant microbe research, because they are time consuming and labor intensive. The development of accurate, high throughput phenotyping platforms will significantly impact our ability to identify promising phenotypes and facilitate hypothesis testing (Mutka and Bart, 2015). We hope that this special focus issue on crop immunity will serve as an important reference for the interaction between plants, pathogens and their biotic environment.

## Conflict of interest statement

The authors declare that the research was conducted in the absence of any commercial or financial relationships that could be construed as a potential conflict of interest.
